# Efficacy of Human Milk Oligosaccharide 6′-Sialyllactose Supplementation on Exercise Performance and Training Adaptations

**DOI:** 10.3390/nu18111743

**Published:** 2026-05-29

**Authors:** Landry Estes, Jacob Broeckel, Nathaniel Rhoades, Giuliet L. Kibler, Ian H. Bivins, Yuhang Liu, Sarah Johnson, Broderick L. Dickerson, Drew E. Gonzalez, Ryan J. Sowinski, Christopher J. Rasmussen, Richard B. Kreider

**Affiliations:** 1 Exercise & Sport Nutrition Lab, Department of Kinesiology and Sport Management, Texas A&M University, College Station, TX 77843, USA; landry.estes@tamu.edu (L.E.); broeckelj@tamu.edu (J.B.); ndrhoades@tamu.edu (N.R.); giulietk@tamu.edu (G.L.K.); ian.bivins@tamu.edu (I.H.B.); yhliu@tamu.edu (Y.L.); sjohnson2216@tamu.edu (S.J.); dickersobl5@tamu.edu (B.L.D.); dg18@tamu.edu (D.E.G.); rjs370@tamu.edu (R.J.S.); crasmussen@tamu.edu (C.J.R.); 2 Department of Kinesiology, School of Natural Sciences, St. Edward’s University, Austin, TX 78704, USA; 3 Occupational, Performance, and Nutrition Lab, Department of Kinesiology, Sam Houston State University, Huntsville, TX 77340, USA

**Keywords:** muscular strength, body composition, anaerobic capacity, aerobic capacity, exercise substrate oxidation, blood lipids

## Abstract

**Background/Objectives**: The purpose of this proof-of-concept study was to examine the effects oligosaccharide 6′-sialyllactose (6′-SL) supplementation (900 mg/d) during training on exercise performance and training adaptations in recreationally active males. **Methods**: In a randomized, double-blind design, 19 healthy males (24.4 ± 6.0 yrs, 174.9 ± 5.9 cm, 82.0 ± 15.2 kg, 27.1 ± 4.7 kg/m^2^, 26.4 ± 6.9% body fat) ingested 3 × 300 mg/d of a placebo or 6′-SL for 12 weeks while partaking in a supervised resistance-training program while following their normal diet. Body composition (DXA), body water, submaximal lactate and substrate oxidation, 5RM dynamic muscular strength, ventilatory anaerobic threshold (VANT), peak aerobic capacity (VO_2_), blood lactate, cycling anaerobic sprint capacity, and fasting blood samples were obtained at week 0, 6, and 12 of training and supplementation. Data were analyzed using multivariate and univariate general linear models (GLM) with repeated measures, along with assessments of mean changes from baseline and corresponding 95% confidence intervals. **Results**: Both groups observed positive training adaptations with no significant differences observed between groups in body composition, 5RM dynamic strength, or anaerobic sprint capacity. Significant interaction effects were observed VANT (*p* = 0.032), VO_2_ at VANT (*p* = 0.028), and submaximal glucose and fat oxidation (*p* = 0.034) while time to reach peak VO_2_ (*p* = 0.083), absolute (*p* = 0.075) and relative (*p* = 0.057) peak VO_2_ approached significance. At Week 6, changes in time to peak effort (196 s [−16, 409], *p* = 0.068), absolute (0.76 L/min [−0.005, 1.53], *p* = 0.051) and relative (10.9 mL/kg/min [0.52, 21.5], *p* = 0.045), and fat oxidation (20.5% [3.1, 37.9], *p* = 0.023) were significantly greater in the 6′-SL group while VANT (−9.2% [−18.3, −0.04], *p* = 0.049), VO_2_ at VANT (−4.8% [−9.8, 0.2], *p* = 0.06) and submaximal glucose oxidation values (−20.5% [−37.9, −3.1], *p* = 0.024) were lower with 6′-SL. After 12 weeks of training, VANT (−9.7% [−17.7, −1.5], *p* = 0.023) and VO_2_ at VANT (−6.4% [−11.8, −1.0], *p* = 0.024) values were significantly lower in the 6′-SL group. No significant differences were observed in resting, submaximal, or maximal exercise blood lactate while the ratios of LDL to HDL (−0.27 [−0.53, −0.01], *p* = 0.042) and total cholesterol to HDL (−0.32 [−0.60, −0.04], *p* = 0.028) decreased significantly from baseline after 6 weeks of training with 6′-SL. **Conclusions**: 6′-SL supplementation did not promote greater gains dynamic strength, fat free mass or changes in body composition. However, while there was some evidence that 6′-SL supplementation influenced training-induced changes in aerobic capacity during the first six weeks, fewer effects were observed after 12 weeks. Moreover, several differences only approached significance in this small proof-of-concept study, so results should be viewed as exploratory and hypothesis generating for additional research.

## 1. Introduction

Human milk oligosaccharides (HMOs), which are present in high concentrations in human breast milk, play a critical role in supporting infant growth and developmental processes [1,2]. Over 130 HMOs have been identified in mature human milk with a number of them purportedly yielding benefits beyond infant growth and development [3]. For instance, reportedly, breast-milk fed infants show a reduced prevalence of diarrhea, and children and adults fed breast milk as infants show reduced occurrence of autoimmune diseases, metabolic syndrome, and food allergies compared to peers fed formula as infants [4,5,6]. Additionally, breastfed infants exhibit a reduced incidence of gastrointestinal and respiratory infections, enhanced cognitive development and function, and reduced risk of becoming overweight or developing obesity [7,8]. Due to their prebiotic properties and their interactions with host immune cell function, HMOS have been explored recently in their roles in human health.

Important biologically active constituents of human milk are sialylated and fucosylated HMOs, with 3′-sialyllatcose (3′-SL) and 6′-sialyllactose (6′-SL) representing most of the total sialic acids present [9]. During the first 100 days of lactation, average concentrations of 3′- and 6′-SL in human milk are approximately 0.19 and 0.64 g/L, respectively with 6′-SL concentrations declining over time [10,11]. Animal models indicate that SL supplementation enhances learning, memory, neuroplasticity, and expression of genes that regulate neural signaling with observable dose-dependent increases in sialylated brain content [12,13,14,15]. Further, sialylated HMOs support microbiota colonization with health-conferring bacteria, such as *Bifidobacterium* and *Bacteroides*, while mitigating colonization of pathogenic genera and promoting production of short-chain fatty acids, thereby contributing to gut health and immune function [1,3,16,17,18]. These studies provide rationale for further investigation of the effects of sialylated HMOs on health outcomes.

Specifically, of the sialylated HMOs, 6′-SL has been reported to possess anti-inflammatory effects and promote immunity, brain development, and gut health [1]. Furthermore, regarding exercise, Spadaro et al. [19] reported that 6′-SL administration increased endurance during simulated swimming in a *Caenorhabditis elegans* (free-living roundworm) model. The authors reported enhanced endurance performance possibly through 6′-SLmediated AMP-activated protein kinase (AMPK) and adenosine receptor signaling. In addition, Park and colleagues [20] administered 6′-SL (100 mg/kg) to C57BL/6J mice for 12 weeks before treadmill testing. Compared to the control, 6′-SL significantly reduced blood lactate levels at rest and post-exercise and increased expression of oxidative enzymes, slow myowin heavy chain (MHC) kinase, and electron transport chain complexes (ETC) I, III, IV, and V of the gastrocnemius muscle fibers, suggesting reduced muscle fatigue and enhanced mitochondrial adaptation. Moreover, the same group reported increased muscle mass and strength of the soleus and gastrocnemius in mice supplemented 100 mg/kg/d for 12 weeks of 6′-SL [21]. However, these studies were conducted in animal models and need validation in human clinical trials before conclusions can be drawn.

In terms of human studies, Kim and workers [22] supplemented healthy adults 3 g/d of 6′-SL for 12 weeks and reported no adverse outcomes or marked changes in clincal safety biomarkers. Also, Park et al. [23] tested 48 weeks of 3–6 g/d of 6′-SL supplementation on GNE myopathy patients and found attenuated fat mass increases in the legs and mitigated hand grip strength declines in the 6′-SL group compared to placebo after 24 weeks of supplementation. The same research group reported significantly increased upper body motor control and decreased fat mass content of the thighs in GNE myopathy patients who supplemented 6 g/d of 6′-SL [24]. No studies have evaluated the effects of 6′-SL supplementation in response to exercise training in healthy individuals. Based on the available basic research and limited human data, we hypothesized that 6′-SL supplementation concurrent to exercise training would improve body composition, exercise performance, and training adaptations. Consequently, we conducted this proof-of-concept clinical trial aimed to determine whether 6′-SL supplementation (900 mg/d for 12 weeks) during resistance training affects exercise performance, body composition, and strength adaptations in men (18–40 years). The primary outcomes were changes in body composition, strength, and blood lactate. Secondary outcomes included changes in aerobic and anaerobic exercise capacity, substrate utilization during exercise, training volume, energy and macronutrient intake, clinical blood panels, and self-reported side effects.

## 2. Methods

### 2.1. Experimental Design

**Figure 1:**
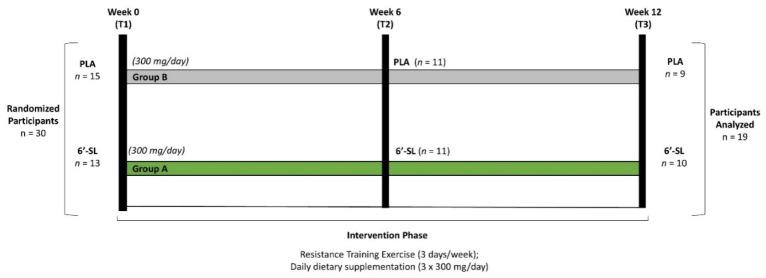
Study timeline. PLA = placebo, 6′-SL = 6′-Sialyllactose.

Experimental design of the study is presented in Figure 1. This trial was conducted as a proof-of-concept, placebo-controlled, parallel-arm, double-blind, counterbalanced study and was performed according to the Declaration of Helsinki standards for the ethical conduct of human research. This study was approved by the Texas A&M University Human Protection Program Institutional Review Board (STUDY2024-1110) and was registered by the ISRCTN—The UK’s Clinical Study Registry (#50097942). The independent variable was 12 weeks of nutritional supplementation of 6′-SL. The primary outcome variables included fat free mass (FFM), fat mass (FM), body fat percentage (BF%), bone mineral content (BMC), bone mineral density (BMD), waist and hip circumferences, and changes in bench press and leg press dynamic strength, and blood lactate concentration. Secondary endpoint variables consisted of peak oxygen consumption, ventilatory anaerobic threshold, submaximal exercise substrate oxidation rates, anaerobic capacity, changes in training volume, energy and macronutrient intake, whole blood biomarkers (cell blood counts), clinical safety markers from serum comprehensive clinical panel (blood lipids, metabolic panel), and self-reported side effects/adverse reactions to supplementation. All data collection and supervised exercise training were conducted in the Exercise and Sport Nutrition Lab (ESNL) within the Human Clinical Research Facility (HCRF) at Texas A&M University. Trained, certified lab personnel supervised all exercise bouts to ensure adherence to general safety practices.

### 2.2. Study Participants

Healthy, recreationally active males between the ages of 18 and 40 years old were recruited to participate in this study. We studied resistance-trained males in this initial study since they are more interested consuming dietary supplements to increase strength and muscle mass during resistance training than other populations. Inclusion criteria included body mass index (BMI) < 40 kg/m^2^ and/or body fat percentage < 35%; recreationally active individuals with at least three months of training history capable of performing whole-body resistance-training and moderate-intensity aerobic exercise without limitations; and willingness to maintain habitual sleep patterns throughout the 12-week intervention period. Participants were not eligible to participate in the study if they planned major changes in lifestyle (i.e., diet, dieting, exercise level, travel) during the study; had no recent history of exercise training; a recent history of more than 5% weight loss; orthopedic limitations that would prevent participation in the training program; uncontrolled heart disease, hypertension, diabetes, thyroid disease, cancer, neurological disease, or untreated psychotic or major depressive disorder; a history of taking muscle-building supplements (e.g., creatine, protein) during the last four weeks or medications that may affect muscle mass or exercise training adaptations; any known allergy to milk protein; and were unable to commit to perform and complete the 12-week supervised training program.

Participants were recruited through local online and print advertisements, campus-wide email bulletins, social media outreach, and flyer postings. Interested individuals completed an initial online or phone eligibility screening questionnaire. Those who met general criteria were scheduled for an in-person familiarization consultation at the research facility. The familiarization included a comprehensive review of study procedures, obtainment of written informed consent, collection of health and medical history, instruction on recording and completing four-day dietary records, and familiarization with exercise testing protocols. Resting vitals (heart rate, blood pressure), height, and weight were also recorded.

**Figure 2:**
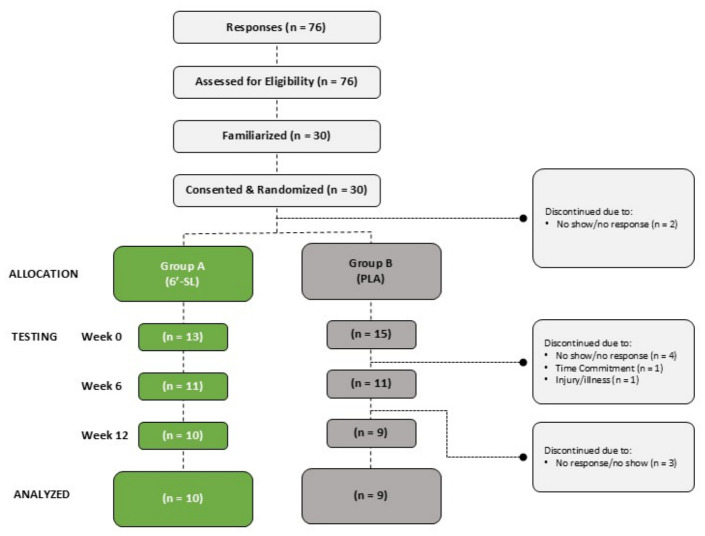
Consolidated Standards of Reporting Trials (CONSORT) diagram. PLA = placebo, 6′-SL = 6′-Sialyllactose.

A Consolidated Standards of Reporting Trials (CONSORT) diagram is provided in Figure 2. Of the 76 individuals who responded to recruitment advertisements, 30 met general inclusion criteria and were enrolled in the study. Of the 30 individuals, 28 participants completed baseline testing and were subsequently randomized into the study groups. Six participants withdrew or were withdrawn after baseline testing for not beginning the training program (4), time commitment issues that limited the ability to train, and 1 from an unrelated injury/illness. A total of 22 participants completed baseline testing, the first six weeks of training, and six-week testing. Thereafter, three participants were withdrawn due to inconsistency in training or not showing up for week 12 testing. Reasons for study withdrawal were non-compliance with training (*n* = 7), study time commitment (*n* = 1), and injury/illness sustained outside of the study procedures (*n* = 1). So, 19 of 24 participants who completed baseline testing and started the training program finished the study (i.e., 79%).

### 2.3. Testing Protocol

**Figure 3:**
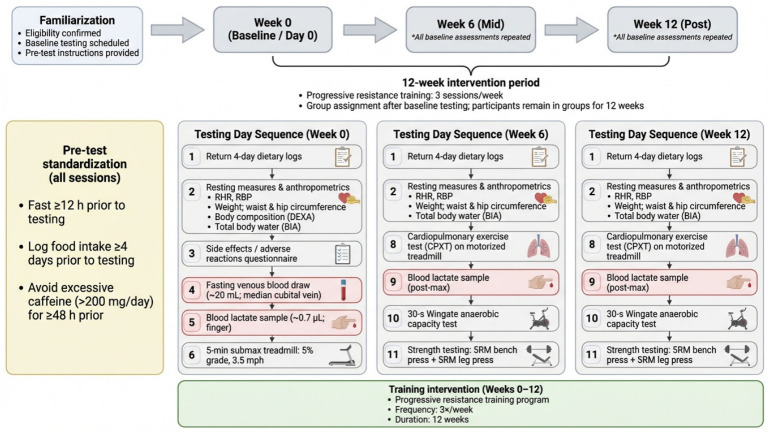
Experimental design and testing sequence across familiarization and Weeks 0, 6, and 12, illustrating standardized pre-test procedures, repeated physiological, metabolic, and performance assessments, and a 12-week progressive resistance training intervention. * = All baseline assessments repeated. Created with FigureLabs.ai and ChatGPT 5.2 (OpenAI, San Francisco, CA, USA).

The testing order of the familiarization and testing sessions conducted at weeks 0, 6, and 12 is shown in Figure 3. At the familiarization session, eligible participants scheduled their baseline testing. They were instructed to fast for at least 12 h prior to all subsequent testing sessions, log food intake for at least 4 days prior to testing, and refrain from excessive caffeine intake (>200 mg/d) for at least 48 h prior to testing. At baseline (day 0), participants returned four-day dietary logs. They had resting measures and anthropometrics assessed, including resting heart rate (RHR) and blood pressure (RBP), weight, waist and hip circumference, body composition via dual-energy X-ray absorptiometry (DEXA), and total body water via bioelectrical impedance analysis (BIA). Subsequently, participants completed a side effects and adverse reactions questionnaire, then donated a fasting venous blood sample (≈20 mL) from the median cubital vein, followed by a blood lactate sample (≈0.7 μL) from a self-selected finger, both being collected using standard procedures. Thereafter, participants completed a 5-min submaximal treadmill test at 5% incline and 3.5 mph, donated another (submaximal) blood lactate sample, and then conducted a cardiopulmonary exercise test (CPXT) on a motorized treadmill. Blood lactate was then collected to assess lactate levels after maximal exercise. Then, participants completed a 30 s Wingate anaerobic capacity test, followed by five-repetition maximum (5RM) dynamic muscular strength tests for bench press and leg press [25,26,27]. Lastly, participants were assigned to groups and remained in them for 12 weeks. All participants completed a 12-week progressive resistance training program three times/wk. All baseline assessments were repeated in weeks 6 and 12 at the same time of day to control for diurnal and cardician rhythm variations.

### 2.4. Familiarization

Those who passed the initial, general screening were invited to the testing facility for an in-person familiarization. During this session, participants reviewed study protocols, provided written informed consent, and provided health and medical histories. They underwent a physical exam during which height, weight, RHR, RBP, and waist and hip circumference measurements were taken. Participants were also familiarized with the exercise testing protocols and provided instructions on recording the four-day diet logs using the smartphone application MyFitnessPal.

### 2.5. Randomization

Allocation to groups was counterbalanced by height, weight, age, and body mass index (BMI) using an adaptive randomization (minimization) method wherein the first participant was assigned to a group with simple randomization, while the subsequent participants were assigned according to their height, weight, age, and BMI in comparison to existing group assignments, with the goal of balancing the averages of these covariates between the two groups [28].

### 2.6. Supplementation and Blinding Protocol

Participants supplemented with either the experimental group, which consisted of 300 mg of the active ingredient (6′-SL, GeneChem, Daejeon, Republic of Korea), microcrystalline cellulose (Comprecel^®^, Mingtai Chemical, Taoyuan City, Taiwan), silicon dioxide (PQ Corp., East Java, Pasuruan, Indonesia), and magnesium stearate (Peter Greven Asia Sdn Bhd., Prai, Menang, Malaysia) or 300 mg of placebo (PLA) containing microcrystalline cellulose, silicon dioxide, magnesium stearate, and sodium chloride. Supplements were shipped as tablets in generic, pre-labeled, blind bottles, labeled by group (A or B) and numerical coding, to ensure double-blind administration. PL was manufactured to resemble the appearance and taste of the experimental group. Both groups were assessed for contaminants and purities by the product manufacturers, who provided certificates of analysis prior to study initiation, verifying the safety of consumption. Participants were instructed to ingest one tablet (300 mg) of their assigned group, three times per day (900 mg/d), with each meal and 8–12 ounces of fluid. Researchers remained blind to the supplement group assignments until after all data was collected and analyzed.

### 2.7. Exercise Training Program

All participants participated in a 12-week, standardized, progressive whole-body resistance training program three times/week. Participants were instructed to report to the testing facility three times per week for supervised exercise training, conducted by certified, trained lab personnel. Participants maintained daily training logs to record their progressive overload of training over the 12 weeks. When participants reported to the exercise training facility for training, they conducted a 5-min warm-up on a treadmill or cycle ergometer, followed by light dynamic stretching. After the warm-up, participants completed 11 upper- and lower-extremity exercises emphasizing all major muscle groups, using machines, free weights, and calisthenics. Exercises included the bench press, seated row, shoulder press, lat pull down, biceps curl, triceps extension, leg press, leg extension, leg curl, abdominal crunches, and back extension. Three sets of 10 repetitions were performed for each movement, with emphasis on increasing resistance by 5% or more every 2 weeks or when comfortably completing all three sets of exercise to ensure adequate increases in training volume. Rest intervals of 2–3 min were incorporated between each set and exercise, and spotters were available if needed. Compliance for the exercise program was set at a minimum of 70% (25/36 exercise bouts), which has been utilized as a benchmark in previous literature [29,30]. Participants who did not meet the minimum 70% compliance, were dropped from the study. Total, upper-body, and lower-body volumes were calculated at the end of the study to track training adaptations. As mentioned above, as a requirement to participate in this study, participants were instructed not to participate in any other structured exercise or recreational activity beyond normal daily activities during the study. Because of this, we did not monitor physical activity beyond the supervised exercise program.

## 3. Procedures

### 3.1. Demographics

A digital sphygmomanometer (Connex^®^ ProBP™ 3400; Welch Allyn, Tilburg, The Netherlands) was used to measure resting heart rate and blood pressure after participants had been seated for at least 5 min. To measure height (cm) and body mass (kg), a self-calibrating digital scale (Health-O-Meter Professional 500KL; Pelstar LLC, Alsip, IL, USA; accuracy ± 0.02 kg) was used. Anthropometric tape measures were used to measure waist and hip circumferences in accordance with standardized measurement protocols to obtain the waist and hip ratios [31,32].

### 3.2. Diet Assessment

Participants used MyFitnessPal (MyFitnessPal, Inc., Baltimore, MD, USA) [33] to log dietary intake four days prior to each testing session. At each time point, four-day food logs (three weekdays, one weekend day) were collected and subsequently analyzed to determine average energy, carbohydrate, protein, and fat intake.

### 3.3. Training Volume Assessment

During supervised training sessions, participants recorded the weight lifted, sets, and repetitions performed to determine training volume the exercises across the 12 weeks. Weight lifted was multiplied by repetitions and sets performed for each exercise to calculate training volume. Training volume of the upper body was calculated by summing the volumes from all upper-body exercises to obtain cumulative volumes for weeks 0 to 6 and 6 to 12. The same method was used to determine cumulative lower-body volume. The cumulative upper- and lower-body volumes were summed to calculate the total volumes for 0–6 and 6–12 weeks.

### 3.4. Body Composition Assessment

Body composition (body mass, FM, FFM, BF%, BMC, BMD), excluding the cranium, was analyzed with a calibrated Hologic Discovery W dual-energy X-ray absorptiometer (Hologic Inc., Waltham, MA, USA) equipped with APEX software version 4.0.2 (APEX Corporation Software, Pittsburgh, PA, USA) [34,35]. In our lab, this assessment has shown a coefficient of variation (Cv) between 0.3% and 0.5% and an average intraclass correlation coefficient (ICC) of 0.98 [36]. Total body water was assessed via multi-frequency bioelectrical impedance analysis (BIA) using the Impedimed SFB7 system (Impedimed, Inc., Carlsbad, CA, USA).

### 3.5. Exercise Assessment

Peak aerobic capacity was captured with the Bruce protocol [37] until volitional fatigue was reached on a motorized treadmill (Trackmaster 425, Newton, KS, USA). A metabolic cart measurement care system (TrueOne 2400, ParvoMedics, Inc., Sandy, UT, USA) was used to capture expired ventilation and oxygen content. A series 5330 calibration syringe (Hans Rudolph Inc., Kansas City, MO, USA) was used to calibrate the pneumotach and the carbon dioxide and oxygen sensors were equilibrated and calibrated to certified, medically-graded gas following manufacturer-recommended procedures. An electrocardiograph (Cardio-Card version 7.2; Nasiff Associates, Brewerton, NY, USA) was employed to monitor heart rate and rhythm, and subjective exertion and fatigue were assessed via the Borg 6–20 Rating of Perceived Exertion (RPE) scale. At the point of volitional fatigue, participants conducted a 10-min cooldown. Ventilatory anaerobic threshold (VANT) was also measured during this assessment. VANT was calculated from a plot of oxygen uptake (VO_2_) against ventilation (V_E_) using previously described methods [38]. Two linear regression lines of fit were positioned to the upper and lower portions of the VO_2_ and V_E_ curves, placed before and after the inflection points. The time point and VO_2_ at which these two lines intersected were defined as the VANT [39]. Submaximal substrate utilization was determined by assessing the average respiratory exchange ratio (VCO_2_/VO_2_) during the last minute of stage II of the Bruce protocol and converting these values to percent carbohydrate and fat oxidation rates [40]. After completing the CPXT, participants conducted a 30 s Wingate anaerobic power cycling assessment using a Lode Excalibur Sport 925900 cycle ergometer (Lode BV, Groningen, The Netherlands) at a standardized work rate of 0.075 kg/kg. Previous research from our lab has shown test-retest reliability for Wingate anaerobic capacity tests, yielding correlation coefficients of r  =  0.98  ±  15% for mean power. Participants then completed a 5RM BP and LP dynamic strength assessment [25,26,27] on a standard bench press and leg press (Nebula Fitness, Versailles, OH, USA) by completing 5RM assessments on both exercises. Five-repetition maximum tests were conducted according to standard procedures, with adequate rest intervals between sets [25,26,27,41]. All testing sessions were performed at the same time of day, in the same order, with same recovery between tests for each participant so any fatigue elicited was consistent from testing session to session.

### 3.6. Blood Collection and Analysis

Fasting venous blood samples from the median cubital vein were collected by certified phlebotomists utilizing previously established procedures [42]. Twenty mL of blood was collected into one ethylenediaminetetraacetic acid (EDTA) tube and two serum separation tubes (SSTs) (BD Vacutainer^®^, Becton, Dickinson and Company, Franklin Lakes, NJ, USA). Blood in the SSTs was allowed to clot at room temperature for 20 min while the EDTA was transported to a 4 °C refrigerator. The SSTs were then centrifuged at 3000× *g* for 10 min at 4 °C using a Thermo Scientific Heraeus MegaFuge 40R centrifuge (Thermo Scientific North America LLC, West Palm Beach, FL, USA). The EDTA and one SST tubes were transported to Clinical Pathology Laboratories, Inc. (Austin, TX, USA; CLIA #45D0505003; CAP Accreditation #21525-01) for complete blood count with differential (whole blood) and comprehensive metabolic panel (serum) analysis. Serum from the remaining SST was aliquoted into microcentrifuge polypropylene tubes (Eppendorf, Enfield, CT, USA) and stored at −80 °C for potential future analysis. In addition, approximately 0.7 μL of arterialized venous blood was taken from a sanitized finger and analyzed for blood located using a calibrated Lactate Plus Meter (Nova Biomedical, Waltham, MA, USA) after the initial venipuncture (resting lactate), post-submaximal treadmill test (submaximal lactate), and post-CPXT (maximal lactate). Intra-analyzer reliability of the analyzer has shown a typical error measurement of 0.4 mM with Cv values at 8.5% [43].

### 3.7. Side Effects

The frequency and severity of side effects (e.g., dizziness, tachycardia, heart palpitations, shortness of breath, blurred vision, nervousness) were assessed using a Likert-type scale, with ratings defined as follows: 0 = none, 1 = minimal (1–2 occurrences per week), 2 = slight (3–4 per week), 3 = moderate (5–6 per week), 4 = severe (7–8 per week), and 5 = very severe (≥9 per week) [29,44]. Participants were additionally encouraged to report any unspecified side effects or adverse reactions they may have experienced in response to their assigned supplementation. This assessment method has demonstrated strong reliability in our laboratory, with coefficients of variation (CVs) ranging from 1.2% to 2.6% [44].

### 3.8. Statistical Analysis

We conducted a comprehensive statistical and practical/clinical analysis with the IBM SPSS Statistics Version 31 software (IBM Corp., Armonk, NY, USA), using procedures previously described in detail [29,45,46,47,48,49,50,51]. Sample size estimates were informed by several considerations. First, our prior studies examining nutritional interventions on exercise performance-related variables with training indicated that 10–15 participants per group were sufficient in a two-group training study to detect significant treatment effects performance-related variables with meaningful magnitude of effects [52,53,54,55,56,57,58,59,60,61,62,63,64,65]. Second, we considered reported means, standard deviations, and statistically significant mean differences from related studies to estimate power, assuming 80% power, variability of approximately 5% to 10% relative to the mean, and expected improvements of 5% to 10% in primary outcomes. Additionally, we obtained funding to do a preliminary pilot study to examine whether results in animals would translate to humans engaged in training. Collectively, we determined that a sample size of ten participants per group in a two-group parallel training designed proof-of-concept or pilot study would be adequately powered to assess meaningful between-group differences. Accordingly, our recruitment goal was to complete twenty participants (10 per group).

Quantitative data were analyzed using mixed model general linear model (GLM) analysis of variance (ANOVA) with repeated measures. Mauchly’s test was used to assess sphericity, and kurtosis statistics were examined to evaluate normality. For variables summarized in tables, omnibus multivariate time, and group × time effects were assessed using Wilks’ Lambda to determine whether the intervention affected the overall set of dependent variables included in the model. Univariate repeated-measures GLM analyses were then used to evaluate intervention effects on individual outcome variables. Greenhouse–Geisser adjusted probability values were reported to control for inflation of the F-statistic when the assumption of sphericity was violated [66,67]. Because of this, the Fisher’s Least Significant Difference (LSD) procedure was used for pairwise comparisons because use of more conservative correction strategies may unnecessarily increase the likelihood of type II error [66,67].

Data were considered statistically significant when the probability of type I error was 0.05 or less. However, because this was a proof-of-concept study and recommendations in sport science have supported interpretation of 90% confidence intervals (CI) equivalent to an alpha level of 0.10 [48,49], we also report outcomes with *p*-values between 0.05 and 0.10 as trends approaching significance. To assist interpretation of these findings, partial eta squared (η_p_^2^) effect sizes were reported to characterize the magnitude of response and to help inform whether additional investigation in larger samples may be warranted. Effect sizes of 0.01 to 0.05 were considered small, 0.06 to 0.13 moderate, and values greater than 0.14 large [68]. Clinical or “practical” significance was evaluated by examining changes from baseline with 95% confidence intervals (CI) [49]. Mean changes with 95% confidence intervals entirely above or below baseline were interpreted as clinically meaningful changes. Fisher’s LSD post hoc analyses were then used to determine whether the magnitude of change differed significantly among groups. We did not incorporate estimates of measurement error or clinician-derived thresholds for minimally meaningful clinical change in this assessment because this was an exploratory dietary supplement pilot study and not designed for clinical use. However, test-retest reliability coefficients of variation are reported to assist readers in judging the practical relevance of the findings.

Data are presented as means ± standard deviations (SD) or as mean changes from baseline expressed as mean change [lower limit, upper limit]. Missing data were minimal and isolated. Missing numerical data, when necessary, were replaced using prior observed value or series means [69], while missing ordinal survey data were substituted using the most frequent response/value method [70]. This approach was considered appropriate because the extent of missingness was very small (<1% of data) and because methodologic literature indicates that replacement of isolated missing values in longitudinal or repeated-measures data should be informed by the participant’s observed response pattern over time and should remain plausible for the specific variable being analyzed, with approaches based on within-subject longitudinal information generally performing better than methods that ignore subject-specific response patterns [71,72,73,74,75]. Thus, our intent was to replace isolated missing observations with values that were representative of the individual’s expected response and unlikely to materially influence statistical inference.

Overall, this analytic framework was designed to provide a thorough and transparent analysis of the data in this proof-of-concept study by integrating omnibus and univariate analyses, effect-size estimates, pairwise comparisons, and change from baseline assessments rather than relying solely on *p*-values [45,46,48]. The statistical methods employed are appropriate and widely accepted for repeated-measures exercise and nutrition studies.

## 4. Results

### 4.1. Demographic Data

Table S1 shows participant descriptive demographic data. Participants were 24.4 ± 6.0 yrs, 174.9 ± 5.9 cm, 82.0 ± 15.2 kg, 27.1 ± 4.7 kg/m^2^, 26.4 ± 6.9% body fat, and had a resting heart rate of 76.4 ± 16.8 beats/min, 129.2 ± 12.6 mmHg resting systolic blood pressure, and 78.6 ± 7.6 mmHg diastolic blood pressure. No significant differences were observed between supplement groups in baseline demographic data.

### 4.2. Macronutrient and Energy Intake

Energy and macronutrient data expressed in absolute (g/d) and relative (g/kg/d) terms were presented in Table S2. Multivariate analysis revealed time (*p* = 0.054, η_p_^2^ = 0.349, large effect) and group × time (*p* = 0.001, η_p_^2^ = 0.485, large effect) within-subject effects. Univariate analysis revealed significant interaction effects (*p* < 0.05) in energy, carbohydrate, and fat intake. Energy intake increased from baseline in the PLA group in response to training while those in the 6′-SL group maintained energy intake. Participants in the PLA group had a greater absolute energy intake compared to the 6′-SL group after Week 6 (335 kcals/d [127, 543], *p* = 0003; 4.67 kcals/kg/d [−0.2, 9.5], *p* = 0.059)) and Week 12 (456 kcals/d [75, 863], *p* = 0.022; 6.69 kcals/kg/d [−0.7, 14.1], *p* = 0.074). Those in the PLA group consumed more carbohydrate at Week 12 (83.9 g/d [36, 131], *p* = 0.002; 1.11 g/kg/d [0.4, 1.8], *p* = 0.003), protein (14.2 g/d [7.8, 26.5], *p* = 0.001; 0.25 g/kg/d [0.07, 0.43], *p* = 0.009), and fat (46.7 g/d [30.4, 63.1], *p* = 0.001; 0.63 g/kg/d [0.22, 1.03], *p* = 0.004) at Week 6 with no differences between groups in protein and fat intake at Week 12. While self-reported energy and macronutrient assessment has limitations, these findings suggest that 6′-SL ingestion during intense training may have some appetite modulating effects.

### 4.3. Training Volume

Table S3 shows lower body, upper body, and total body training volume data expressed in absolute and relative terms. No significant differences were observed between groups in supervised training session compliance (*p* = 0.865). Multivariate analysis showed a significant time (*p* = 0.005, η_p_^2^ = 0.286, large effect) with no group × time (*p* = 0.110, η_p_^2^ = 0.182, large effect) within-subject effects. Univariate analysis revealed that the training program promoted 2–3 fold significant increases over time in lower body (*p* = 0.001, η_p_^2^ = 0.928, large effect; *p* = 0.001, η_p_^2^ = 0.147, large effect), upper body (*p* = 0.001, η_p_^2^ = 0.934, large effect; *p* = 0.001, η_p_^2^ = 0.935, large effect), and total lifting volume (*p* = 0.001, η_p_^2^ = 0.933; *p* = 0.001, η_p_^2^ = 0.933, large effect) expressed in absolute and relative terms, respectfully. A significant interaction effect was observed in absolute lower body lifting volume (*p* = 0.023, η_p_^2^ = 0.203, large effect) while relative values approached significance (*p* = 0.074, η_p_^2^ = 0.147, large effect) with significantly greater volume observed in the 6′-SL group at Week 12 (80,288 kg [13,749, 146,826], *p* = 0.021; 764 kg/kg [107, 1421], *p* = 0.025). A significant interaction effect was also observed in total lifting volume expressed in absolute terms with changes greater in the 6′-SL group compared to PLA at Week 12 (107,114 kg [4134, 210,094], *p* = 0.042) while relative changes approached significance (921 kg/kg [−144, 1987], *p* = 0.086). No significant interaction effects were observed in remaining training variables. These findings show that both groups responded well to the training program with some evidence of a greater training volume at Week 12 in the 6′-SL group.

### 4.4. Body Composition

**Figure 4:**
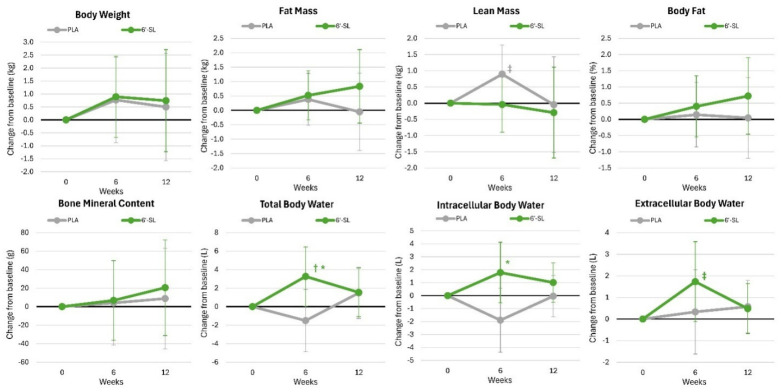
Changes in body composition and body water-related variables. PLA = placebo. 6′-SL = 6′-Sialyllactose. † = *p* < 0.05 (‡ *p* > 0.05 to *p* < 0.10) difference from baseline. * = *p* < 0.05 difference between groups.

Table S4 presents body composition and hydration-related variables. No significant multivariate time (*p* = 0.811, η_p_^2^ = 0.165, large effect) or group × time (*p* = 0.518, η_p_^2^ = 0.220, large effect) within-subject effects were observed. Univariate analysis revealed significant interaction effects in total body water (*p* = 0.038, η_p_^2^ = 0.178, large effect) with intracellular water approaching significance (*p* = 0.067, η_p_^2^ = 0.159, large effect). Pairwise comparisons showed that participants in PLA group observed a significant increase in lean tissue mass (0.896 kg [0.00, 1.79], *p* = 0.05) while total body water (3.26 L [0.07, 6.45], *p* = 0.045) and extracellular water (1.73 L [−0.12, 3.58], *p* = 0.065) increased in the 6′-SL group. Changes from baseline with 95% CIs are shown in Figure 4. This analysis revealed that changes in total body water (4.76 L [0.13, 9.39], *p* = 0.045) and intracellular water (3.69 L [0.28, 7.08], *p* = 0.036) were significantly greater in the 6′-SL group at Week 6 but not Week 12.

### 4.5. Anaerobic Training Adaptations

**Figure 5:**
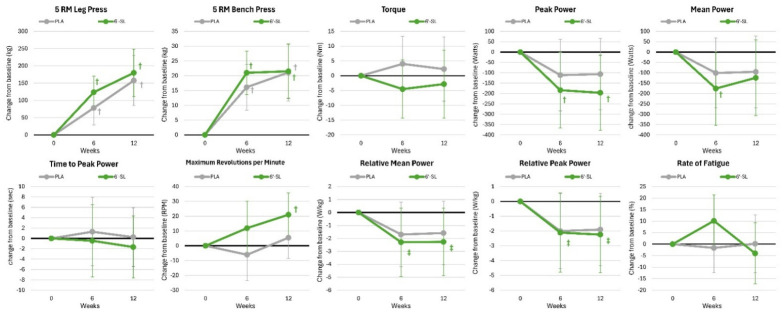
Changes in anaerobic training-related variables. PLA = placebo. 6′-SL = 6′-Sialyllactose. RM = repetition maximum. † = *p* < 0.05 (‡ *p* > 0.05 to *p* < 0.10) difference from baseline.

Table S5 shows muscular endurance and anaerobic power-related variables. Multivariate analysis revealed significant time (*p* < 0.001, η_p_^2^ = 0.725, large effect) and group × time (*p* = 0.009, η_p_^2^ = 0.480, large effect) within-subject effects indicating a significantly different response to training adaptations between groups. Univariate analysis found significant time effects in 5RM leg press (*p* = 0.001, η_p_^2^ = 0.718, large effect) and bench press (*p* = 0.009, η_p_^2^ = 0.684, large effect), while peak power (*p* = 0.017, η_p_^2^ = 0.243, large effect) and mean power (*p* = 0.045, η_p_^2^ = 0.208, large effect) decreased from baseline. However, no significant univariate interactions or pairwise comparison differences were observed between groups aft 6 and 12 weeks of training. Mean changes analysis from baseline are presented in Figure 5. This analysis also found an overall time (*p* < 0.001, η_p_^2^ = 0.725, large effect) and interaction effects (*p* = 0.009, η_p_^2^ = 0.480, large effect) with apparent differences in changes in 5RM dynamic muscular strength and endurance (both groups), peak power, mean power, and revolutions per minute during the sprint test. However, no significant pairwise comparisons differences were observed between groups at Week 6 and 12.

### 4.6. Aerobic Training Adaptations

**Figure 6:**
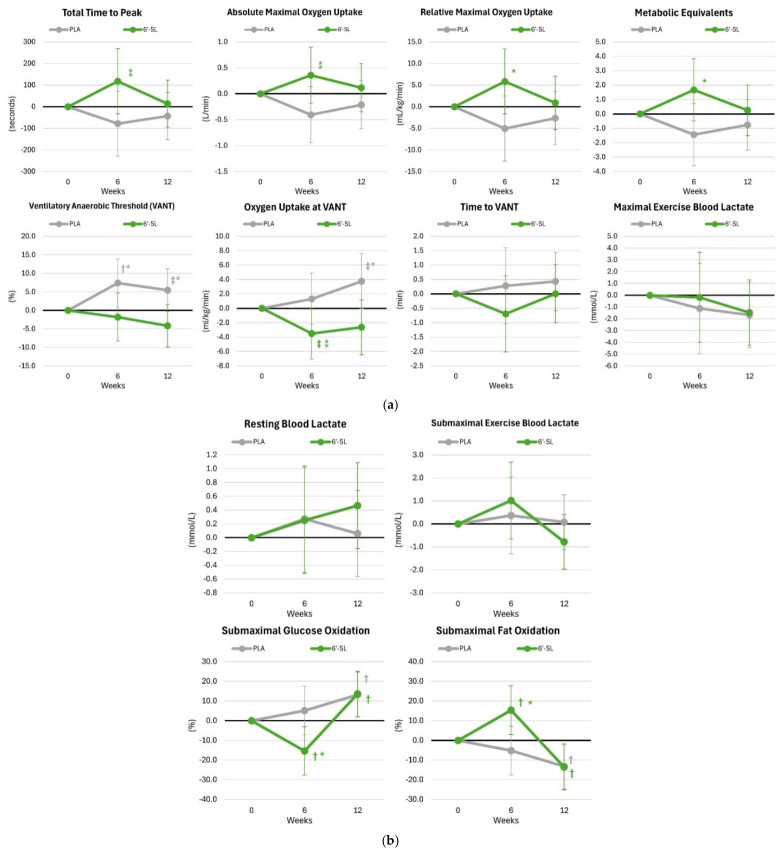
(**a**) Changes in aerobic training-related variables. (**b**) Changes in aerobic training-related substrate-utilization variables. PLA = placebo. 6′-SL = 6′-Sialyllactose. † = *p* < 0.05 (‡ *p* > 0.05 to *p* < 0.10) difference from baseline. * = *p* < 0.05 (⁑ *p* > 0.05 to *p* < 0.10) difference between groups.

Table S6 shows aerobic training adaptations and resting, submaximal, and maximal lactate results. Multivariate analysis revealed no significant time (*p* = 0.046, η_p_^2^ = 0.476, large effect) or group × time (*p* = 0.277, η_p_^2^ = 0.380, large effect) within-subject effects. Univariate analysis revealed significant interaction effects in ventilatory anaerobic threshold (VANT, *p* = 0.032, η_p_^2^ = 0.201, large effect), VO_2_ at VANT (*p* = 0.028, η_p_^2^ = 0.209, large effect), and submaximal glucose (*p* = 0.034, η_p_^2^ = 0.196, large effect) and fat oxidation (*p* = 0.034, η_p_^2^ = 0.196, large effect) while time to reach peak VO_2_ (*p* = 0.083, η_p_^2^ = 0.151, large effect), absolute (*p* = 0.075, η_p_^2^ = 0.154, large effect) and relative (*p* = 0.057, η_p_^2^ = 0.171, large effect) peak oxygen uptake, and metabolic equivalents (METS, *p* = 0.057, η_p_^2^ = 0.170, large effect) values approached significance. No significant interaction effects were observed between groups in time to VANT or resting, submaximal, or maximal lactate levels. Figure 6a,b show changes from baseline in these variables. Similar interaction effects were noted as described when analyzing mean changes from baseline with significant or data approaching significant increases after Week 6 in time to peak (196 s [−16, 409], *p* = 0.068), absolute (0.76 L/min [−0.005, 1.53], *p* = 0.051) and relative (10.9 mL/kg/min [0.52, 21.5], *p* = 0.045), maximal METS (3.1 [0.07, 6.2], *p* = 0.045), and fat oxidation (20.5% [3.1, 37.9], *p* = 0.023) in the 6′-SL group while VANT (−9.2% [−18.3, −0.04], *p* = 0.049), VO_2_ at VANT (−4.8% [−9.8, 0.2], *p* = 0.06) and submaximal glucose oxidation values (−20.5% [−37.9, −3.1], *p* = 0.024) were lower with 6′-SL. After 12 weeks of training, VANT (−9.7% [−17.7, −1.5], *p* = 0.023) and VO_2_ at VANT (−6.4% [−11.8, −1.0], *p* = 0.024) values were significantly lower in the 6′-SL group. Lactate levels were non-significantly affected after 6 and 12 weeks of training with 6′-SL.

### 4.7. Cell Blood Counts

Table S7 shows whole blood cell counts. Multivariate analysis found no significant time (*p* = 0.139, η_p_^2^ = 0.519, large effect) or group × time (*p* = 0.449, η_p_^2^ = 0.438, large effect) within-subject effects. Univariate analysis showed no significant interaction effects among variables, except for eosinophils (*p* = 0.022, η_p_^2^ = 0.214, large effect) and granulocytes (*p* = 0.083, η_p_^2^ = 0.141, large effect). Pairwise comparisons found significant differences between groups at week 6 in red cell dimension width, neutrophils, lymphocytes, monocytes, and eosinophils. However, these differences were small, within measurement error, not seen at Week 12, and well-within normal clinical values.

### 4.8. Markers of Catabolism

Table S8 shows markers of catabolism from the comprehensive metabolic panel. Multivariate analysis found no significant time (*p* = 0.776, η_p_^2^ = 0.226, large effect), but significant group × time (*p* = 0.039, η_p_^2^ = 0.427, large effect) within-subject effects. Univariate analysis did not reveal any time or group-by-time interaction effects for these variables. Pairwise analysis revealed differences between groups in aspartate aminotransaminase at week 6 and 12 and creatinine at week 12.

### 4.9. Glucose and Blood Lipids

**Figure 7:**
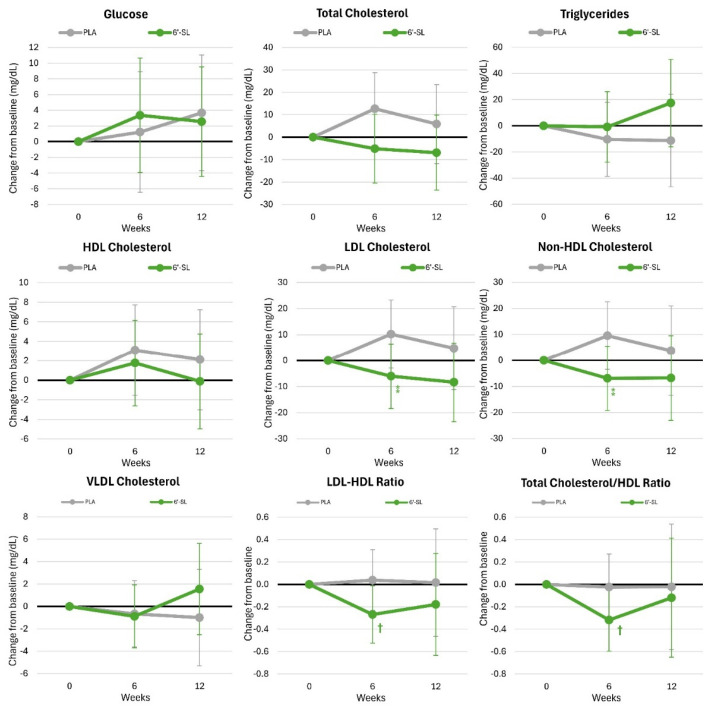
Changes in glucose and blood lipids. PLA = placebo. 6′-SL = 6′-Sialyllactose. † = *p* < 0.05 difference from baseline. ⁑ *p* > 0.05 to *p* < 0.10 difference between groups.

Table S9 presents glucose and blood lipid results. Multivariate analysis showed no significant time (*p* = 0.862, η_p_^2^ = 0.129, medium effect) or group × time (*p* = 0.926, η_p_^2^ = 0.110, medium effect) within-subject effects. Similarly, no significant univariate time or interaction effects were observed in glucose or blood lipid-related variables. Pairwise analysis revealed that the ratios of LDL to HDL (−0.27 [−0.53, −0.01], *p* = 0.042) and total cholesterol to HDL (−0.32 [−0.60, −0.04], *p* = 0.028) decreased significantly from baseline after 6 weeks of training in participants following 6′-SL. However, no significant differences were observed between groups in these variables. Figure 7 shows the mean changes from baseline. The ratio of LDL to HDL and total CHL to HDL values decreased from baseline with 6′-SL after 6 weeks of supplementation and training. Additionally, changes in LDL (−16.2 mg/dL [−34.3, 1.8], *p* = 0.075) and non-HDL cholesterol (−16.5 mg/dL [−34.4, 1.5], *p* = 0.069) approached significant differences between groups at week 6.

### 4.10. Serum Electrolytes

Table S10 shows serum electrolyte results. Multivariate analysis showed significant time (*p* = 0.008, η_p_^2^ = 0.314, large effect), but no group × time (*p* = 0.765, η_p_^2^ = 0.098, medium effect) within-subject effects. Univariate analysis revealed no significant group × time interaction effects. Pairwise analysis indicated that sodium levels increased after 12 weeks with 6′-SL with carbon dioxide decreasing in both groups. However, these differences were small, within measurement error, and well-within normal clinical values.

### 4.11. Side Effect Analysis

Table S11a,b present the reported side effect frequency and severity. Chi square analysis found no significant differences between groups in the frequency or severity of side effects. Side effects noted were typically infrequent and of minimal severity. No study withdrawals due to any reported side effects in response to supplementation occurred over the 12 weeks.

## 5. Discussion

This exploratory proof-of-concept study aimed to investigate whether daily supplementation of 900 mg·d^−1^ 6′-SL for 12 weeks during a progressive resistance training program influenced body composition and training adaptations in men aged 18–40 years. The primary findings indicate that 6′-SL supplementation did not enhance resistance training-induced gains in lean mass or maximal strength after 12 weeks relative to placebo. However, 6′-SL supplementation significantly increased intracellular body water and tended to increase extracellular body water after six weeks. Supplementation was also associated with reduced energy and carbohydrate intake. Although no improvements in anaerobic training adaptations were noted, improvements in aerobic capacity and greater fat oxidation were reported after six weeks. In addition, more favorable changes in LDL cholesterol and LDL/HDL, total cholesterol/HDL ratios after six weeks of 6′-SL supplementation were found. While results of this exploratory study should be interpreted with caution and viewed as preliminary and hypothesis generating in nature, results provide some support to contentions that 6′-SL supplementation may have some effects on exercise training adaptations. The following provides additional insight to results observed.

### 5.1. Primary Outcomes: Body Composition and Resistance Training Adaptations

Recent evidence from animal models have suggested that 6′-SL administration augments endurance and strength-type exercise performance via enhanced muscle volume and size. For example, Park and colleagues [21] evaluated chronic oral 6′-SL supplementation in male C57BL/6J mice using a 100 mg·kg^−1^·d^−1^ dose for 12 weeks and reported improved grip strength, running distance, time to exhaustion, total work, and greater tissue weights of the gastrocnemius and soleus muscles. Accompanying the increased muscle weights was also increased total MHC protein expression. In a secondary analysis, the same authors also found 6′-SL treatment increased slow MHC isoforms in the gastrocnemius muscle, upregulation of ETC I, III, IV, and V complexes, and blunted rises in blood lactate concentration after exhaustive treadmill exercise, which all contributed to improved exercise performance [20]. In a separate experiment, Park and workers [23] also supplemented GNE myopathy patients 6 g·d^−1^ of 6′-SL for 48 weeks and showed enhanced hand grip power and an attenuated increase in fat accrual of the thighs; however, muscle thickness was not assessed.

Due to prior evidence suggesting interactions with muscle and strength, a primary objective of this investigation was to determine whether 6′-SL supplementation augmented resistance training-induced alterations in body composition and strength. In this experiment, despite significant time effects reflecting expected training adaptations in both groups, no significant between-group differences were observed in body mass, fat mass, lean mass, maximal strength, or measurements of muscular power assessed during the Wingate. Both groups increased training volume across the 12 weeks; however, the 6′-SL group experienced a significant increase in total lifting volume. Nevertheless, the greater training volume did not lead to significant differences between groups in lean tissue mass or strength adaptations. The discrepant findings between the current study and previous animal studies could be attributed to species differences, increased dosages used in previous animal models and randomized clinical trials, and/or training stimuli. The current study subjugated healthy young males to a progressive resistance training program while previous literature that expressed positive muscle-related outcomes did not incorporate training or use endurance-based training.

Conversely, 6′-SL yielded significantly increased total and intracellular fluids with a trend toward increased extracellular fluid at six weeks suggesting a cellular hydration effect and redistribution of fluid compartments. 6′-SL-induced increases in total body fluid compartments is a novel finding that has not been shown in previous literature. Typically, resistance training increases lean tissue concomitantly to total body water and intracellular fluid [76,77,78]. Although both groups similarly increased total body weight and fat free mass, only 6′-SL experienced significant increases in total body water and intracellular water at six weeks, which were significantly higher than PLA values. However, these fluid shifts were not accompanied by greater fat-free mass accrual compared to placebo. In this regard, 6′-SL might influence cellular hydration status via osmotic regulation. Since cellular hydration influences protein synthesis, more research is needed to explore how ingesting HMOs might impact fluid distribution dynamics with and without exercise training. Nevertheless, present findings indicate that 6′-SL supplementation at 900 mg·d^−1^ for 12 weeks does not exert changes in body composition or yield strength-enhancing effect when combined with resistance training in healthy young men.

### 5.2. Secondary Outcomes: Aerobic Capacity, Substrate Utilization, and Metabolic Adaptations

Previous evidence has suggested augmented endurance exercise performance in animals treated with 6′-SL. Spadaro et al. [19] treated Caenorhabditis elegans with 0.2, 1, and 2 mg/mL 6′-SL and found dose-dependent improvements in swimming exercise performance due to possible morphological adaptations in mitochondria, altered AMPK and adenosine receptor signaling, and changes in substrate utilization from fat to glucose. Additionally, Park and workers [20,21] reported enhanced treadmill exercise performance in rodents due to enhanced mitochondrial adaptations, increased expression of type I MHCs, and reduced blood lactate concentrations at rest and post-exercise indicating reduced fatigue. Stoian and Mănescu [79] also recently suggested that substrate availability may influence crosstalk between AMP-activated protein kinase (AMPK) and mechanistic target of rapamycin (mTOR) and thereby exercise fuel utilization and training adaptations. In this regard, these researchers proposed a Training-Fuel Coupling (FTC) model where training during low-energy and low-glycogen states may activate AMPK-dependent pathways supporting oxidative efficiency while training in a well-fed state may facilitate mTOR-mediated protein synthesis [79]. Collectively, these reports provide some plausibility that nutritional strategies that affect AMPK may influence substrate utilization and/or training adaptations.

In the present study, individuals in the 6′-SL group experienced a significant increase in relative peak oxygen uptake from baseline while absolute tended to increase at six weeks, though these improvements returned to baseline levels by week 12. The findings at six weeks are similar to Spadaro et al. [19] and Park et al. [20,21] who noted significant improvements in endurance exercise in their animals treated with 6′-SL. Conversely, participants in the PLA group experienced a significant increase in VANT while 6′-SL yielded no change expressed relative to peak oxygen uptake. Oxygen uptake at VANT tended to decrease in the 6′-SL participants, who also experienced marked reductions in glucose oxidation with a simultaneous significant increase in fat oxidation at six weeks. Cumulatively, these findings suggest that 6′-SL may influence early metabolic adaptations to training, particularly those related to substrate utilization and aerobic capacity. Our findings of increased fat oxidation disagree with that of Spadaro et al. [19] who noted animals treated with 6′-SL exhibited significantly lower muscle glycogen levels and decreased fat oxidation post-exercise compared to control animals. Last, resting and submaximal blood lactate concentrations remained unchanged in both groups. These findings disagree with that of Park and researchers [20] who reported significantly lower resting and post-exercise blood lactate concentrations after 6′-SL in rodents. The differences in outcomes between our study and previous studies utilizing animal models might be attributed to species disparities, 6′-SL dosage, diet, and/or laboratory methods. Given the small sample size of this pilot study and number of comparisons evaluated, the possibility of Type I statistical error should also be considered. Nonetheless, the observed increase in aerobic capacity and fat oxidation after 6 weeks of 6′-SL supplementation is interesting and deserves additional study to assess whether 6′-SL supplementation during endurance training may influence AMPK signaling, peroxisome proliferator-activated receptor gamma coactivator-1α (PGC-1α) [80], and/or other makers mitochondrial function [81].

### 5.3. Dietary Intake and Cardiometabolic Markers

Participants receiving 6′-SL reported lower total energy intake, with a concomitant reduction in carbohydrate but increase in fat intake, despite no prescribed dietary intervention during an intense exercise program. Although there are known limitations in evaluating self-reported diet records (e.g., under reporting, reporting bias), these findings are interesting and suggest that 6′-SL may have suppressed appetite during intensified training. These dietary differences may have also influenced training-induced changes in fat oxidation observed. While the mechanisms underlying this finding cannot be determined from the present data, HMOs are known to interact with gut-immune and host–microbiota pathways that influence metabolic regulation [1,2]. More specifically, recent evidence has shown 6′-SL administration, combined with fucosylated HMOs, to mice increased microbial diversity and enriched beneficial taxa, such as *Bacteroides,* while impairing potentially unfavorable genera, such as *uncultered_rumen_bacterium* and *Ruminococcus* [82]. These findings raise the possibility that 6′-SL may influence appetite regulation or substrate preference. Given present findings, additional research in humans using controlled feeding designs and assessing mechanistic endpoints, such as appetite hormones, microbiota, gut function, etc. may be warranted.

Additionally, significant reductions in lipid ratios (LDL:HDL, total cholesterol:HDL) with moderate reductions in LDL and non-HDL cholesterol were observed with 6′-SL supplementation. These findings align with previous studies showing blunted increases in blood lipid concentrations in rodents fed a high-fat diet with HMOs [83,84]. Reductions in LDL cholesterol observed in the 6′-SL group might be explained by enhanced sialylation of LDL particles, which has been shown to reduce atherogenicity through improved lipid clearance [83,85,86]. Additionally, sialylated HMOs modulate intestinal lipid metabolism and genes involved in cholesterol transport and inflammation (*Apoe*, *Apob*, *Pltp*, *Hmgcs2*) [83,87,88,89,90,91]. These findings suggest that 6′-SL may have relevance for cardiovascular health, particularly when evaluated in populations with dyslipidemia or during structured weight-loss interventions, although larger randomized, placebo-controlled, clinical trials are needed to substantiate these claims.

### 5.4. Safety and Tolerability Compared with Human Trials

No significant differences in supplementation-related side effects were observed between treatment conditions, and no participants withdrew due to adverse events, indicating that 6′-SL supplementation was well tolerated. Although our sample size was small, these findings are consistent with prior human trials. Kim et al. [22] supplemented healthy adults with 3 g of 6′-SL for 12 weeks and reported no clinically meaningful adverse effects or changes in standard clinical chemistry markers. Further, Park and colleagues reported no adverse events associated with 6′-SL supplementation over 48 weeks [23] and 96 weeks [24] in GNE myopathy patients. Regulatory evaluations have similarly concluded that 6′-SL is safe for use as a novel food ingredient under proposed conditions of use [92]. In addition, extensive toxicological evaluations in rodents have demonstrated high safety margins for 6′-SL sodium salt, including lack of genotoxicity and a high No Observed Adverse Effect Level in dosing studies [93]. These data support the safety and tolerability of 6′-SL supplementation in humans across a range of doses and contexts. However, the lack of significant differences may also be related to the small sample size evaluated. Therefore, more research is needed in larger clinical trials to evaluate safety markers before conclusions can be drawn.

### 5.5. Limitations and Future Directions of Research

Though results are interesting, there are several limitations that need to be considered when interpreting results of this proof-of-concept study. First, the sample size was modest, which may have limited statistical power to detect small-to-moderate differences in between-group effects in some variables. While we observed a number of significant differences, several differences only approached significance (*p* > 0.05 to *p* < 0.10) with moderate to large effect sizes that may have been significant if a larger sample size was studied. It is also possible that we may have missed some findings that may have been significant if a larger sample was evaluated. Moreover, since we evaluated a number of variables, it is possible that some variables were significant by chance to do the number of comparisons evaluated. Consequently, results should be interpreted with caution and viewed as preliminary and hypothesis generating for additional research. Second, this study assessed the effects of 12 weeks of 900 mg/d of 6′-SL supplementation. Most differences were observed after 6 weeks but not 12 weeks of supplementation and training. It is possible that higher doses supplemented over longer periods of time might confer greater benefits. Third, we did not measure inflammatory or oxidative stress makers so this discussion should be viewed speculatively based on interpretation of prior research. Fourth, dietary intake was self-reported, introducing a potential for reporting error and/or bias. Although there was some evidence that 6′-SL supplementation suppressed energy intake in response training, we did not assess appetite hormones or gut microbiota composition and function, so these findings need to be interpreted as speculative. Fifth, the training program emphasized resistance exercise, thereby limiting endurance exercise adaptations that may have been observed with a training program that focused more on endurance training. Sixth, since we only evaluated a small sample of young resistance trained males willing to take the supplements and participate in an intense 12-week supervised training program, results may not be generalizable to females, older individuals, or untrained populations. Finally, although no side effects or clinically significant changes in standard markers of health were observed suggesting that the supplementation protocol was well-tolerated, the sample size was small so additional research is needed to assess safety.

With that said, present findings from this proof-of-concept study provide some support to contentions that 6′-SL supplementation may have some benefit for individuals engaged in intense training and warrants additional research with a larger sample size. Future research should: (1) evaluate the effects of 6′-SL administration in conjunction with modest energy deficit (e.g., 300–500 kcal/d) and greater endurance exercise training volume on endurance exercise outcomes; (2) evaluate the effects of longer supplementation periods with higher daily dosages of 6′-SL on training adaptations and markers of health; (3) evaluate the effects of 6′-SL supplementation in females, older individuals, and untrained populations; (4) assess the effects of 6′-SL supplementation on inflammatory and oxidative stress markers; (5) evaluate how 6′-SL affects appetite hormones and gut microbiota in healthy individuals; and (6) determine if 6′-SL supplementation with and without a standardized exercise program can elicit cognitive function benefits.

## 6. Conclusions

In summary, within the limitations of this preliminary pilot study, 6′-SL supplementation (900 mg·d^−1^ for 12 weeks) during a standard progressive resistance training program appeared to be well tolerated but did not enhance gains in lean mass or maximal strength in healthy young men. However, supplementation was associated with favorable changes in dietary intake patterns, aerobic capacity, submaximal exercise fat oxidation, and lipid ratios, particularly after 6 weeks of training, consistent with prior mechanistic and preclinical evidence describing the metabolic effects of sialylated HMOs [1,19,20,22]. These findings support further investigation of 6′-SL as a metabolic-support ingredient in exercise and weight-management interventions but not as a muscle mass or strength-enhancing supplement. However, results should be interpreted with caution and viewed as preliminary and hypothesis generating for additional research.

## Data Availability

The original contributions presented in this study are included in the article/Supplementary Material. Further inquiries can be directed to the corresponding author.

## Supplementary Materials

The following supporting information can be downloaded at: https://www.mdpi.com/article/10.3390/nu18111743/s1, Table S1: Participant descriptive data, Table S2: Energy and macronutrient intake, Table S3: Training volume, Table S4: Body composition and body water results, Table S5: Anaerobic training adaptations, Table S6: Aerobic training adaptations, Table S7: Whole blood cell counts, Table S8: Markers of catabolism, Table S9: Glucose homeostasis and lipids, Table S10: Serum electrolytes, Table S11a: Frequency of side effects, Table S11b: Severity of side effects.

## Abbreviations

The following abbreviations are used in this manuscript:

3′-SL	3′-Sialyllactose
6′-SL	6′-Sialyllactose
AMPK	AMP-activated protein kinase
BF%	Body Fat Percentage
BIA	Bioelectrical Impedance Analysis
BMC	Bone Mineral Content
BMD	Bone Mineral Density
BMI	Body Mass Index
BP	Bench Press
CI	Confidence Interval
CONSORT	Consolidated Standards of Reporting Trials
CPXT	Cardiopulmonary Exercise Test
Cv	Coefficient of Variation
DEXA	Dual-Energy X-ray Absorptiometry
EDTA	Ethylenediaminetetraacetic Acid
ESNL	Exercise and Sport Nutrition Lab
ETC	Electron Transport Chain
FFM	Fat Free Mass
FM	Fat Mass
GLM	General Linear Model
HCRF	Human Clinical Research Facility
HDL	High-Density Lipoprotein
HMO	Human Milk Oligosaccharide
HR	Heart Rate
ICC	Intraclass Correlation Coefficient
LDL	Low-Density Lipoprotein
LL	Lower Limit
LP	Leg Press
LSD	Least Significant Difference
METs	Metabolic Equivalents
MHC	Myosin Heavy Chain
PLA	Placebo
PGC-1α	Peroxisome Proliferator-Activated Receptor Gamma Coactivator-1α
RBP	Resting Blood Pressure
REE	Resting Energy Expenditure
RHR	Resting Heart Rate
RM	Repetition Maximum
RPE	Rating of Perceived Exertion
SA	Sialic Acid
SST	Serum Separation Tube
TG	Triglycerides
UL	Upper Limit
VANT	Ventilatory Anaerobic Threshold
VE	Ventilation
VO_2_	Oxygen Uptake
VCO_2_	Volume of Expired Carbon Dioxide
